# Total rupture of hydatid cyst of liver in to common bile duct: a case report

**DOI:** 10.11604/pamj.2014.19.370.5727

**Published:** 2014-12-10

**Authors:** Hassan Robleh, Fahmi Yassine, Khaiz Driss, Elhattabi Khalid, Bensardi Fatima-zahra, Berrada Saad, Lefriyekh Rachid, Fadil Abdalaziz, Zerouali Ouariti Najib

**Affiliations:** 1 Service des Urgences Chirurgie Viscérale, Centre Hospitalier Universitaire Ibn Rochd, Casablanca, Morocco

**Keywords:** Hydatid liver cyst, Rupture, Common bile duct (CBD), jaundice

## Abstract

Rupture of hydatid liver cyst into biliary tree is frequent complications that involvethe common hepatic duct, lobar biliary branches, the small intrahepatic bile ducts,but rarely rupture into common bile duct. The rupture of hydatid cyst is serious life threating event. The authors are reporting a case of total rupture of hydatid cyst of liver into common bile duct. A 50-year-old male patient who presented with acute cholangitis was diagnosed as a case of totally rupture of hydatid cyst on Abdominal CT Scan. Rupture of hydatid cyst of liver into common bile duct and the gallbladder was confirmed on surgery. Treated by cholecystectomy and T-tube drainage of Common bileduct.

## Introduction

Hydatid disease is a health problem still causingconcern in endemic areas such as the Mediterraneanregion [1]. Inhumans, 50% to 75% of hydatid cysts occur in the liver,25% are found in the lungs, and 5% to 10% are distributedalong the arterial system [2]. Hydatid cysts of the liver exert pressure on the surrounding parenchyma, and in approximately one-fourth of the cases, due to higher pressure in the cyst, the cysts eventually leak into small bile ducts or perforate into large ones [3]. The most common complication of hydatid cyst of the liver is spontaneous rupture into the biliary tract with biliary obstruction being reported to occur in 5% to 17% of cases [3]. Liver hydatid can rupture in any part of biliary system but the communication with the hepatic bile ducts is most common. Rupture between a hepatic hydatid cyst and the gallbladder is rare [4]. Intrabiliary rupture occurs into the right duct in 55-60% of cases, into the left duct in 25-30% and rarely into the confluence or gall bladder [5]. We present a case of a hydatid cyst of the liver which ruptured spontaneously into the common bile duct resulting in jaundice and cholangitis thattreated on emergency cholecystectomy,T-tube drainage of common bile duct after clearance of cyst daughters and their membranes from the common bile duct.

## Patient and observation

**Figure 1:**
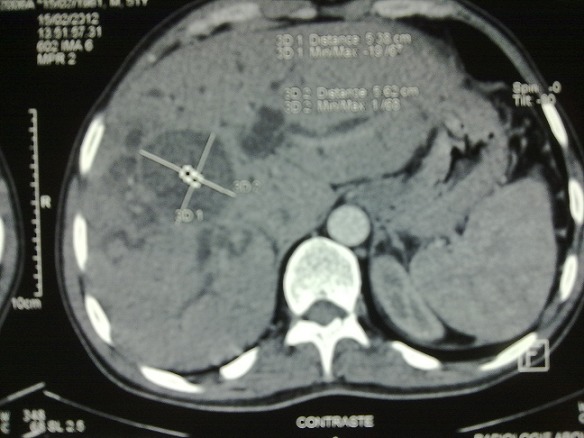
Abdominal CT showing hydatid cyst of 4th & 5th segments of liver communicating wit dilated CBD

**Figure 2:**
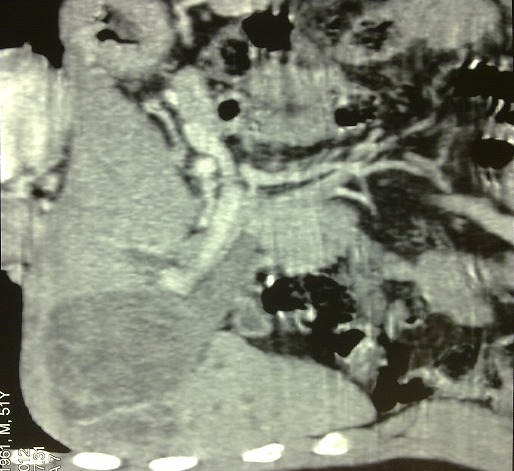
Abdominal CT showing hydatid cyst of 4th & 5th segments of liver communicating wit dilated CBD

A fifty-year-old male with no special note of past or social history presented with high-grade fever and right hypochondrial pain of two week duration. The patient was jaundiced, febrile and had a tender hepatomegaly just below right costal. Investigations showed a deranged liver function test (LFT) with a total bilirubin of 258,40mg/dl, AST(420 U/L), ALT(415 U/L), G.G.T(328 UI/L) and raised serum alkaline phosphatase(1181 U/L). Hydatid serology was positive. CT scan revealed intraparenchymic hydatid cyst type IV(4th and 5th segments) that opened totally into common bile duct (Figure 1, Figure 2).

**Figure 3:**
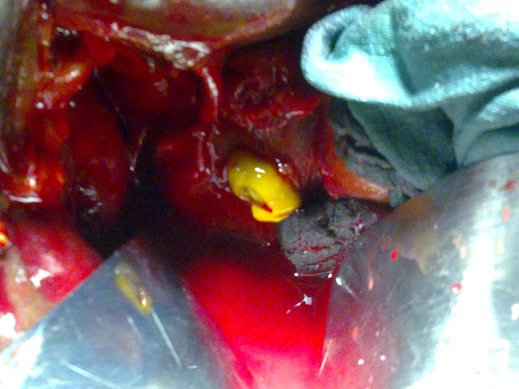
Operative view showing a hydatid materiel of CBD after choledochotomy

The cyst was seen to communicate inferiorly with common hepatic duct in the region of ductal confluence with extension of the hydatid membranes into common hepatic duct (CHD) and proximal CBD withupstream dilatation of the right and left hepatic ducts and intra-hepatic biliary radicles with ruptured membranes present, the right intrahepatic duct was seen communicating with cyst cavity. CBD was dilated with internal debris seen inside and Gallbladder was distended showing irregular linear intensities inside. Atretic hydatid liver cyst containing infected bile with ruptured membranes into common bile duct through probably right hepatic duct (Figure 3). Patient under went right subcostal laparotomy was performed, cholecystectomy and choledochotomy with evacuation of cystic content and debris via CBD with irrigation with 0.9% NaCl solution, T-tube drainage of CBD and under hepatic drainage by Salem tube. The postoperative period was unremarkable and patient was discharged on day 7, Salem's tube was removed on day 6thand the T-tube was removed after a control cholangiography was done on day 16th postoperatively.

## Discussion

Hepatic hydatid disease after a long asymptomatic course becomes symptomatic in an unknown percentage of patients. Hydatid cysts grow at a variable rate and stabilize, and may become calcified, while others may collapse and completely resolve. The clinical course is long and even after surgical treatment; decontamination is difficult to achieve [6]. The rupture of the hydatid cyst is probably the most common complication of liver hydatid disease, appearing in about 15% of all cases [7]. Cysts of the liver exert pressure on the surrounding parenchyma, and in most cases the cysts eventually rupture into the biliary tree in 1% to 25% of cases although an incidence of 64.75% has been reported from a multicentric study in Tunisia [3]. Ultrasonography and CT have been reported to be the main diagnostic methods, with 85% and 100% sensitivity, respectively, in identifying hydatid cyst rupture [2]. It is difficult to suspect the diagnosis pre-operatively. In most of the reported cases, the diagnosis has been made only at operation, the pre-operative diagnosis being calculus jaundice, amoebic liver abscess, cholangitis, empyema of gall bladder or carcinoma of the head of the pancreas [8]. In the present case,the primary symptom was jaundice with fever. Therefore, we diagnosed as acute angiocholitis due to calculus or rupture of hydatid cyst into biliary tree that confirmed on abdominal CT scan.

The most effective procedures following the evacuation of hydatid material from the biliary tree remaincontroversial. Alper et al argued that wide choledochoduodenostomy decreases morbidity and mortality, whereas Ulualp et al and Humayun et al considered Kehr drainage adequate. The groups of Lygidakis, Paksoy, and Kornaros preferred T-tube drainage and performed choledochoduodenostomy in less than 10% of cases. Lygidakis et al. reported one death among the patients who underwent T-tube drainage (3.4%), but none of their patients experienced external fistula. In a series of 64 patients, Daali et al. reported only four cases (6.3%) of prolonged biliary drainage [1]. In this case report, he had cholecystectomy and T-tube drainage of the common bile duct after its clearance the cyst membranes and debris with verification of its permeability. According our experience,T-tube drainage is superior to choledochoduodenostomy

## Conclusion

Hydatid hepatic cyst rupture into the biliary tree is frequent common complication of hydatid disease. Usually, it leads to biliary colic, cholangitis and jaundice. Accurate diagnosis and emergency surgical intervention is mandatory, if there is suspect or an obvious biliary communication with the liver cyst.
